# Dendritic Cell Immunoreceptor Is a New Target for Anti-AIDS Drug Development: Identification of DCIR/HIV-1 Inhibitors

**DOI:** 10.1371/journal.pone.0067873

**Published:** 2013-07-09

**Authors:** Alexandra A. Lambert, Arezki Azzi, Sheng-Xiang Lin, Geneviève Allaire, Karianne P. St-Gelais, Michel J. Tremblay, Caroline Gilbert

**Affiliations:** 1 Centre de recherche du CHU de Québec Infectious Diseases and Immunology, Quebec, Canada; 2 Endocrinology and Genomics (Faculty of Medicine), Université Laval, Quebec, Canada; 3 Department of pharmacology, College of Medicine, Al-Imam Mohammad Ibn Saud Islamic University (IMSIU), Riyadh, Saudi Arabia; Imperial College London, United Kingdom

## Abstract

The HIV-1 pandemic continues to expand while no effective vaccine or cure is yet available. Existing therapies have managed to limit mortality and control viral proliferation, but are associated with side effects, do not cure the disease and are subject to development of resistance. Finding new therapeutic targets and drugs is therefore crucial. We have previously shown that the dendritic cell immunoreceptor (DCIR), a C-type lectin receptor expressed on dendritic cells (DCs), acts as an attachment factor for HIV-1 to DCs and contributes to HIV-1 transmission to CD4^+^ T lymphocytes (CD4TL). Directly involved in HIV-1 infection, DCIR is expressed in apoptotic or infected CD4TL and promotes trans-infection to bystander cells. Here we report the 3D modelling of the extracellular domain of DCIR. Based on this structure, two surface accessible pockets containing the carbohydrate recognition domain and the EPS binding motif, respectively, were targeted for screening of chemicals that will disrupt normal interaction with HIV-1 particle. Preliminary screening using Raji-CD4-DCIR cells allowed identification of two inhibitors that decreased HIV-1 attachment and propagation. The impact of these inhibitors on infection of DCs and CD4TL was evaluated as well. The results of this study thus identify novel molecules capable of blocking HIV-1 transmission by DCs and CD4TL.

## Introduction

The discovery of new therapeutic targets and the development of new therapeutic approaches are necessary in order to pursue the fight against **h**uman **i**mmunodeficiency **v**irus type 1 (HIV-1). The drugs currently available or in development for treating HIV-1 infection target the virus itself and its replication mechanisms and thus risk selecting resistant variants. Although these treatments increase the lifespan of patients, they also contribute to increased co-morbidity [1]. Studies of a simian model and more recently of human HIV-1 show that treatment during the acute phase of infection improves the immune response to the virus [2], [3]. It has been demonstrated that early events in HIV-1 infection are highly determinant in the irreversible damage inflicted to key immune cells [3], [4], [5], [6], [7]. To maintain vital immune competency, it is crucial to find new targets involved in the first steps of viral transmission and prevent the devastating initial damage to the immune system.

The first immune cells to establish contact with invading HIV-1 are dendritic cells (DCs), which then communicate with cells of both the innate and adaptive immune systems [8], [9]. DCs are intricately involved in the initial response to HIV-1- [9], [10], [11]. During primary infection, HIV-1 in mucosal tissue is first internalized by DCs, which then migrate to secondary lymphoid organs, where the virus is transferred to CD4+ T lymphocytes (CD4TL). Translocation of internalized virus appears to occur via a cell-to-cell junction (the so-called virological synapse) created by simple physical contact between DC and CD4TL [12], leading to virion production in both cell types. Transfer of HIV-1 from DCs to CD4TL occurs in two distinct phases [13], [14], [15]. During the initial phase, virus located within endosomal compartments of DCs is transported to the intercellular junction and then internalized by CD4TL. A later second phase is dependent on productive infection of DCs and storage of viral progeny. We have recently demonstrated that the C-type lectin receptor known as dendritic cell immunoreceptor or DCIR [16] allows HIV-1 to attach to DCs and enhances HIV-1 infection in both phases [17], unlike DC-SIGN (dendritic cell-specific intercellular adhesion molecule-3-grabbing non integrin), which is only involved in the early phase [18], [19]. Among the various HIV-1 cell surface receptors expressed in DCs, only DCIR has been shown to play a key role in viral dissemination, initiation of infection [17] and antiviral immunity [20]. Furthermore, it is very likely that interaction between DCIR and HIV-1 is a major factor in HIV-1 pathogenesis since DCIR expression in CD4TL is induced by HIV-1 or by apoptosis as we have previously shown [21]. CD4TL apoptosis is an indicator of HIV-1 pathogenesis in both the early and later phases of AIDS. In view of DCIR expression on DCs and its role in HIV-1 transmission *in vitro*, this receptor holds promise as a target for preventing HIV-1 infection and possibly decreasing HIV-1 transmission during the chronic phase of the disease, in which CD4TL apoptosis increases.

DCIR is expressed primarily in cells of the myeloid lineage (i.e. neutrophils, DCs, monocytes and macrophages) as well as in B cells [16]. In addition, interaction between DCIR and HIV-1 is likely of significance in HIV-1 pathogenesis since we have observed DCIR expression in HIV-loaded CD4TL both in vitro and from HIV-1-infected patients [21], as well as in apoptotic CD4TL. However, the physiological functions of DCIR are not fully understood. DCIR has been associated with some auto-immune diseases [22]. DCIR was detected at the surface of plasmacytoid DCs [23] and may regulate DC expansion [22]. In myeloid or plasmacytoid DCs, internalization of DCIR inhibits the response of TLR8 or TLR9 [23], [24], two Toll-like receptors known to play an important role in innate immunity against viruses.

DCIR is the product of the human gene *CLEC-4A,* which encodes a protein 237 amino acid residues in length and is unique among the lectin receptors due to the presence of several unique structural motifs. It contains an intracellular signalling consensus sequence known as ***i*** *mmunoreceptor* ***t*** *yrosine-based* ***i*** *nhibition* ***m*** *otif* or ITIM [25], a *neck domain* important for HIV-1 binding that includes a carbohydrate recognition domain (CRD) extracellular portion [26], and an EPS motif (Glu-Pro-Ser), that is, a specific galactose recognition domain [25]. We have determined that the ITIM motif is required for DCIR-mediated enhancement of HIV-1 infection [27]. Furthermore, we have shown, using antibodies directed against the EPS motif or CRD domain, or by deleting the neck domain, that these extracellular portions are both involved in the binding of HIV-1 and its subsequent transfer to CD4TL [17].

Given this potentiation of HIV infection through interaction with DCIR, our objective was to develop a molecule to inhibit HIV binding to DCIR. Considering that the virus-encoded viral envelope glycoprotein gp120 is one of the most heavily glycosylated proteins known in nature (reviewed by Vigerust and Shepherd [28]) and that DC-SIGN-dependent HIV-1 capture requires interaction between gp120 and the CRD domain of DC-SIGN [18], [29], it might be that a similar interaction allows DCIR to act as an attachment factor for HIV-1. The EPS motif of DCIR is known to bind specifically to galactosyl residues of glycoproteins [30]. Since galactosyl residues are present on the surface of HIV-1, we designed and synthesized chemical inhibitors targeting the EPS and/or CRD domains of DCIR.

Virtual screening has recently helped to discover ligands and inhibitors based on crystallographic [31] and homology models of target proteins [32], [33]. Studies have shown that virtual docking to homology models frequently yields enrichment of known ligands as good as that obtained by docking to a crystal structure of the actual target protein [32], [34], [35]. This structure-based approach to inhibitor design has been used to identify several inhibitors of 17β-hydroxysteroid dehydrogenases [36], [37], [38] and RNA-dependent RNA polymerase [39]. Methodical analysis of the structure of DCIR is required to design potent and specific inhibitors of its interaction with HIV-1, via the CRD and/or EPS motifs, thereby generating potential new drugs. Since no complete or partial tertiary structure has been published for DCIR, we built a homology model using the structure of the CRD of CLEC4M ( = L-SIGN), which also interacts with gp120, as a template.

Based on this model, several inhibitors were selected using virtual screening and tested using various methods. This study shows that specific chemical inhibitors directed against the EPS motif or CRD domain of DCIR prevent the attachment of HIV-1 to DCs and to apoptotic or infected CD4TL, without any side effect on CD4TL proliferation. Our DCIR homology model, in addition to providing detailed structural information, will help in the development of new lead compounds using virtual screening combined with *in vivo* testing.

## Materials and Methods

### Reagents

IL-4 was purchased from R&D systems (Minneapolis, MN) and granulocyte-macrophage colony-stimulating factor (GM-CSF) was purchased from Genscript (Piscataway, NJ). Cells were grown routinely in RPMI 1640 medium supplemented with 10% fetal bovine serum (FBS), penicillin G (100 U/ml), streptomycin (100 µg/ml), L-glutamine (2 mM) – all purchased from Wisent BioProducts (St-Bruno, QC, Canada) and with primocin (Amaxa Biosystems, Gaithersburg, MD, USA). The culture medium for HEK293T cells was made of DMEM (Invitrogen, Burlington, ON, Canada) supplemented with 10% FBS, penicillin G (100 U/ml), streptomycin (100 µg/ml), and L-glutamine (2 mM).

### Molecular Modeling of DCIR

We modeled human DCIR structure using the SWISS MODEL homology modeling program via their website interface [40], [41], run in automatic mode after submitting the DCIR sequence (UniProtKB/Swiss-Prot accession number: Q9UMR7). The resulting model was examined using the PDB viewer program [42].

### Docking Site Selection and Pre-docking Preparation

The 3D model of DCIR served as a receptor for the docking studies. Hydrophobic pockets on the DCIR extracellular domain structure (residues 103–233) were evaluated with the Site Finder application under the MOE program (Chemical Computing Group, Montreal, QC, Canada) and ranked according to their hydrophobic contacts and locations. The protein model was inspected visually for accuracy of the χ_2_ dihedral angles of Asn and His residues and the χ_3_ angle of Gln, and was rotated 180° as needed to maximize hydrogen bonding. The proper histidinyl tautomer was selected manually to maximize hydrogen bonding. Finally, all aspartyl, glutamyl, arginyl and lysyl residues were treated usually as charged species.

Ligand docking on the DCIR was carried out using Genetic Optimization Ligand Docking (GOLD) version 3.3 [43]. Ligand and side-chain flexibility was allowed during docking. The ChemScore scoring function as implemented in the GOLD program was used to estimate the change in free energy that occurs upon ligand binding to a protein: ChemScore = Δ*G_binding_+P_clash_ +c_internal_ P_internal_+(c_covalent_ P_covalent_ +P_constraint_)*, with Δ*G_binding_* = Δ*G_o_*+Δ*G_hbond_ S_hbond_*+Δ*G_metal_ S_metal_*+Δ*G_lipo_ S_lipo_*+Δ*G_rot_ H_rot_*, where *P_clash_, P_internal_*, *P_covalent_* and *P_constraint_* are penalty factors included to prevent poor geometries in docking due respectively to atom positions, internal torsion constraint, valence-angle bending and other constraints (*c_internal_* and *c_covalent_* being scale factors for the respective penalty terms). *S_hbond_*, *S_metal_*, and *S_lipo_* respectively are the free energy scores accounting for the contributions of hydrogen bonding, acceptor-metal and lipophilic interactions to ligand-protein interaction, whereas *H_rot_* is a score representing the loss of conformational entropy of the ligand upon binding to the protein. Δ*G_hbond_*, Δ*G_metal_*, Δ*G_lipo_*, and Δ*G_rot_* are regression coefficients derived from multiple linear regression analysis on a training set of 82 protein–ligand complexes from the PDB [44].

### Virtual Screening

The free public database ZINC 8 compiles over two million compounds [45]. A subset of 128,000 compounds with drug-like properties and satisfying the Lipinski Rule of Five [46] were selected for further analysis. The database was used for virtual screening for the selected docking sites of DCIR and compounds were ranked according to their ChemScore [44] and hydrogen bonding potential.

### Antiviral Compounds

The seven top-scoring compounds identified by virtual screening as potential inhibitors of HIV-1 attachment to DCIR were purchased from Sigma-Aldrich (Oakville, ON, Canada) or ChemBridge (San Diego, CA) for *in vitro* screening using our DCIR-expressing cell model. A stock solution of each compound in 10 mM in DMSO was prepared, and inhibitor concentrations up to 10 µM were tested. This concentration was judged reasonable, given our goal of developing a lead compound with therapeutic potential, from which molecules efficacious at even lower concentrations presumably could be derived. The results presented in this manuscript were obtained using inhibitor at 10 µM and a final DMSO concentration of 0.1%.

### Cells and Viral Stocks

Human embryonic kidney (HEK) 293T cells were cultured in DMEM supplemented with 10% FBS. The Raji-CD4 cell line is a B cell line carrying the Epstein-Barr virus and rendered susceptible to HIV-1 infection by stable transfection with cDNA encoding human CD4 [47]. Raji-CD4 cells stably expressing DCIR (Raji-CD4-DCIR) were obtained following retroviral transduction as described previously [48]. In some experiments, we also used Raji-DC-SIGN, that is, Raji cells stably transfected with a plasmid encoding DC-SIGN [49]. Primary human DCs were generated from purified human monocytes (i.e. CD14^+^ cells). Peripheral blood was obtained from healthy donors. CD14^+^ cells and CD4^+^ T cells (CD4TL) were then isolated from fresh PBMCs using a monocyte-positive selection or negative selection kit according to the manufacturer’s instructions (MACS CD14 microbeads, STEMCELL Technologies, Vancouver, BC, Canada) as described previously [14], [15], [48]. Cells were solicited from anonymous, healthy volunteer donors who had signed an informed consent approved by the CHUL research ethics review board. NL4-3 (X4) and NL4-3/Bal*env* (R5) were produced upon transient transfection of HEK293T cells as described previously [14], [15], [48].

### Virus Binding/Entry and Infection Assays on Raji-CD4-DCIR Cells

Where indicated, 1×10^6^ parental Raji-CD4 cells (DCIR-negative), Raji-CD4-DCIR or Raji-DC-SIGN transfectants were pre-treated with the indicated amount of a chemical inhibitor for 10 min. Cells were then pulsed with NL4-3 as described previously [48].

### HIV-1 Binding and Virus Infection Assays on iMDDCs

For assessing binding/entry, iMDDCs (3×10^5^ cells in a final volume of 300 µl) were pre-treated with 10 µM of different chemical inhibitors for 10 min and exposed to NL4-3/Bal*env* (30 ng of p24) for 60 min at 37°C. After three washes with PBS, cells were re-suspended in PBS containing 1% BSA. The HIV capsid particle p24 content was determined by ELISA, while susceptibility of iMDDCs to HIV-1 infection was assessed by initially exposing 3×10^5^ cells to NL4-3/Bal*env* (30 ng) at 37°C for 2 h. After washes, cells were maintained in complete RPMI 1640 supplemented with GM-CSF and IL-4 in 96-well plates, in a final volume of 200 µl. Every three days over a nine-day period, half of the conditioned medium was collected and kept at −20°C until assayed. Virus production was estimated as above by measuring p24 levels by ELISA.

### HIV-1 Binding/Entry, Infection and Transfer Experiments with CD4TL

Apoptosis was induced in PHA-L/IL2-activated CD4TL treated with 30 µM H_2_O_2_ for 16 h before performing the following experimental procedures. To assay binding/entry, cells (1×10^6^) were incubated for 60 min at 37°C with NL4-3 (100 ng of p24). After three thorough washes with PBS to remove un-adsorbed virus, HIV-1 binding was quantified by estimating p24 content. For the transfer studies (or trans infection), H_2_O_2_-treated CD4TL (1×10^6^) were incubated with NL4-3 (100 ng of p24) for 2 h and after washes, autologous PHAL/IL-2-activated CD4TL (1×10^6^) were added (ratio 1∶1) in complete RPMI 1640 medium supplemented with rhIL-2 (30 U/ml). Every other day, half of the conditioned medium was collected and kept at −20°C and the culture replenished with fresh medium. For all studies, virus production was estimated after 3 days of co-culture by measuring the p24 levels in cell-free culture supernatants.

### Statistical Analysis

Statistical analyses were carried out according to the methods outlined by Zar [50] and Sokal and Rohlf [51] using the GraphPad Prism software. Means were compared using two-tailed Student’s *t-*tests, or a single-factor ANOVA followed by Dunnett’s multiple comparisons when more than two means were considered. Each graph included at least three experiments performed in triplicate. The data are presented with SEM in order to hi-light differences from the mean. SEM also takes sample size into account. *P* values <0.05 were deemed statistically significant. Asterisks denote statistically significant differences (*P<0.05, **P<0.01, ***P<0.001).

## Results

### Molecular Modeling of DCIR

**Figure 1:**
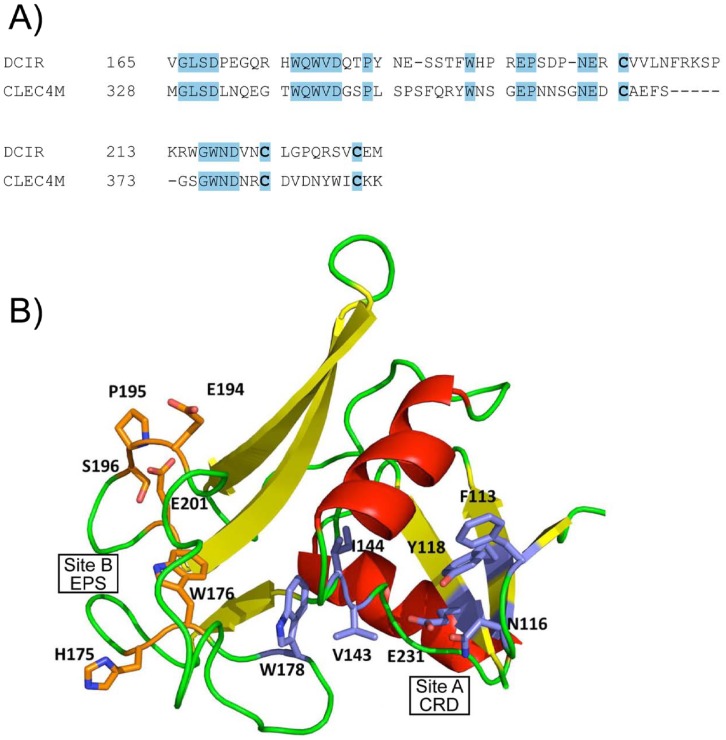
DCIR modelling. **A) Partial sequence alignment** between CLEC4M (GenBank™ #AAI10615) and DCIR (GenBank™ # NP_057268) indicating completely conserved regions and residues. **B)** **Three-dimensional model** showing positions of the selected docking sites on the dendritic cell immunoreceptor (DCIR) model (103-233) for virtual screening runs according to the examples. Residues forming site A are represented by the *blue* portions while *orange* indicates those forming site B.

To identify inhibitors that could prevent HIV-1 attachment to the extracellular domain of DCIR, we used a virtual template or homology model, based on lectin structure. CLEC4M (**DOI:**10.2210/pdb1sl6/pdb), a gp120-binding lectin also known as CD299 or L-SIGN, has a crystal structure corresponding to a fragment of DC-SIGNR (a homolog of DC-SIGN expressed in epithelial cells) in a complex with CD15 (3-fucosyl-*N*-acetyl-lactosamine) and thus appeared best suited for this purpose [52]. A model of the carbohydrate-binding domain defined by residues 166 to 233 of DCIR was built from CLEC4M and refined using the SWISS MODEL website interface. About 33% of the amino acid residues of this model correspond to the DCIR sequence, as shown in Figure 1A. The proposed tridimensional model has four beta sheets and two alpha helices, as illustrated in Figure 1B.

### Virtual Screening and Molecule Selection

**Figure 2:**
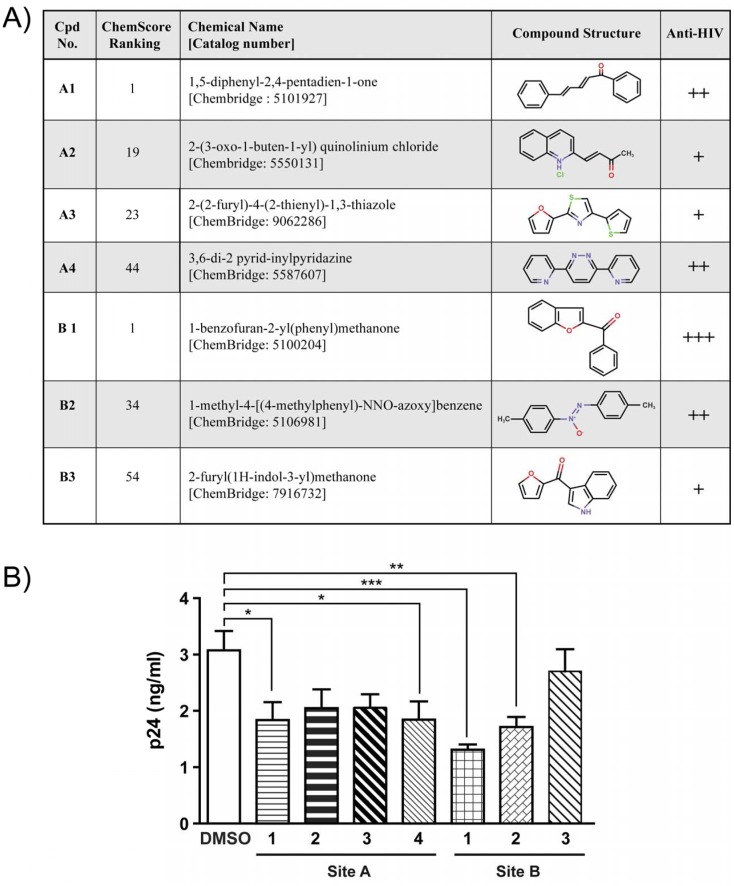
Selected compounds screened and tested *in vitro*. **A)** Chemical structure of potential inhibitors of HIV-1 attachment to DCIR, selected by virtual screening, Compounds selected from CRD site are depicted as A1 to A4. Those selected from EPS site are named as B1 to B3 Virtual interacting compounds with both site of DCIR modeling have anti-HIV activity. **B)** Inhibitors decrease HIV-1 attachment to DCIR: Raji-CD4-DCIR cells were treated with one of four site-A inhibitors or one of three site-B inhibitors (inhibitor concentration = 10 µM) or with solvent (10 mM DMSO) only for 10 min at 37°C. Cells were thereafter exposed to NL4-3 virus for 60 min. After three washes with PBS to remove un-adsorbed virus, the abundance of cell-associated virions was quantified by measuring p24 content. Data correspond to mean ± SEM of three independent experiments performed with triplicate samples. Asterisks (*) denote statistically significant values (*, *P*<0.05, ** P<0.01, *** P<0.001).

Docking sites for inhibitors of HIV-1 binding were selected from a number of potential sites identified by the Site Finder utility of the Molecular Application Environment (MOE) program (cf. “Materials and Methods”). The primary targeted sites are two hydrophobic pockets. Site A (associated with the CRD domain) is located near the surface and is delimited by DCIR residues Phe113, Asn116, Tyr118, Val143, Ile144, Trp178 and Glu231, while site B (associated with the EPS motif), nearly on the opposite side of the structure, is delimited by residues 194-198 plus His175, Trp176 and Glu201 (Figure 1B). Virtual screening of inhibitory compounds was performed for site A within a sphere delimited by a 10-Å radius around the OE1 atom of residue Glu231 and for site B within a sphere delimited by a 10-Å radius around the NE1 atom of Trp176. The top 100 compounds as ranked by the ChemScore function in the GOLD docking program were selected for visual inspection of their docking orientations. The four compounds selected for site A and the three for site B are represented in Figure 2A. All chemical products identified were readily available commercially for *in vitro* tests. Using specific antibodies, we had previously surmised the importance of CRD and EPS in the process of HIV-1 attachment [17].

### CRD and EPS Inhibitors Decrease HIV-1 Attachment

For rapid estimation of the capacity of inhibitors targeting these structures to alter the initial steps in HIV-1 biology, Raji-CD4 cells stably transfected with DCIR (Raji-CD4-DCIR) were used as described previously [27]. Figure 2B shows a statistically significant decrease in HIV-1 attachment to cells pre-treated with inhibitors A1, A4 and B1 and B2. In contrast, inhibitors A2, A3 and B3 are the least efficient blockers of HIV-1 attachment as indicated in panel A. These latter three were therefore excluded from further study in order to focus our attention on four molecules A1, A4, B1 and B2 for complete validation in more physiological model of primary cells.

### Determination of Specificity and Toxicity of Inhibitors

**Figure 3:**
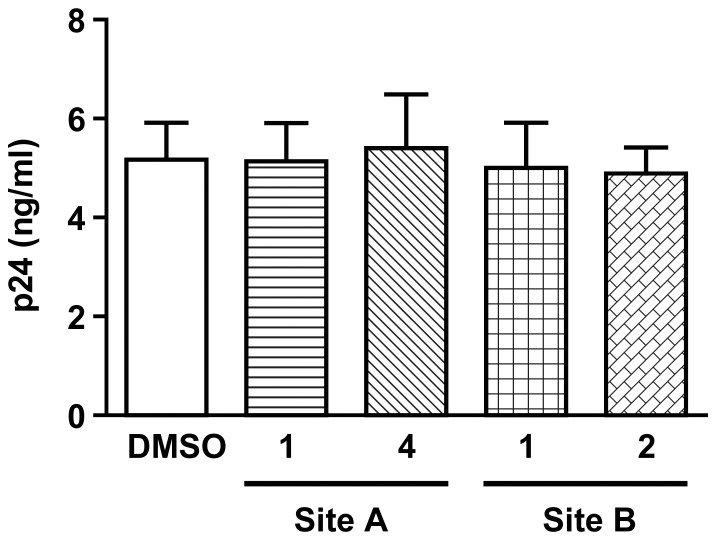
DCIR inhibitors do not affect other C-type lectin binding such as DC-SIGN. Raji-DC-SIGN cells were treated for 10 min at 37°C with inhibitors 1 and 4 directed against site A and 1 and 2 directed against site B, or with DMSO. Afterwards, cells were pulsed with NL4-3 for 60 min, rinsed thrice with PBS to remove un-adsorbed virus, and cell-associated viruses were quantified by measuring p24 content. Data shown correspond to the means ± SEM of 3 independent experiments performed in triplicate.

DC-SIGN is also known to play an active role in HIV-1 binding and transfer by DCs [18]. To assess the specificity of these inhibitors and whether they interacted with other C type lectins, their effects on HIV-1 binding to Raji-DC-SIGN cell lines were tested. Figure 3 shows that these inhibitors do not affect HIV-1 binding to DC-SIGN. These data are consistent with the selected inhibitors being specific for DCIR and inactive with DC-SIGN, despite the fact that both lectins are C-type and closely related.

**Figure 4:**
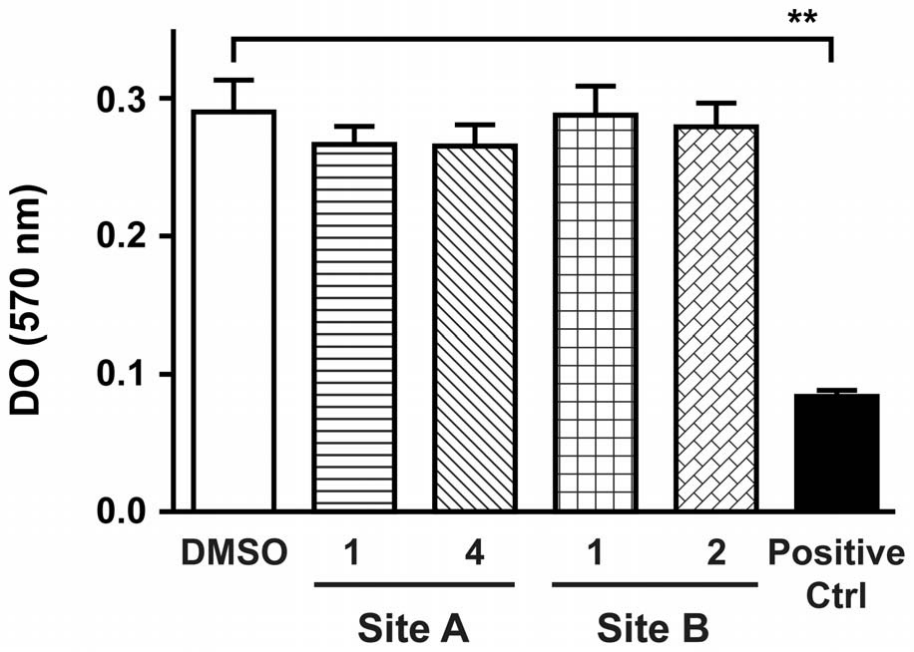
Impact of inhibitors on lymphocytes proliferation. PBMC (2×10^5^ cells/200 µl) were pre-incubated with DCIR inhibitors (10 µM) (or vehicle), or with a toxic molecule as a positive control at 100 uM (Ctrl) before mitogenic stimulation with PHA-L/IL-2 (1 µg/ml and 30 U/ml). Cell proliferation was stopped at day 3 by adding MTT reagent and SDS as described in “Materials and Methods”. *A*_570_ was then determined. Data shown correspond to the means ± SEM of 3 independent experiments performed in triplicate. Asterisks denote statistically significant values (**, *P*<0.01).

It was also crucial to determine the impact of these inhibitors on cellular viability. Peripheral blood mononuclear cells (PBMCs) were pre-incubated with the inhibitors before measuring mitogenic stimulation and cellular proliferation using the MTT method. Figure 4 shows clearly that the inhibitors did not affect cell proliferation and that the observed, for all cell lines or primary cells used in this study, decrease in HIV-1 binding induced by these compounds resulted from disrupted interaction with DCIR and was not a mere consequence of reduced viability (Data not shown). Dose response was also performed and toxicity was observed for concentration over 50 µM for all four inhibitors (data not shown). Potential side effects of these inhibitors on cells expressing a large amount of DCIR was tested, and the results show that neutrophil functional response such as de-granulation was not affected by these inhibitors (data not shown). This observation strengthens the conclusion that these inhibitors seem specific and not toxic. Finally, the impact of the inhibitors on the expression HIV-1 receptor CD4 or co-receptors CCR-5 and CXCR4 on DCs was assessed by cytofluorometry and their expression was not affected by inhibitors pre-treatment (Data not shown).

### DCIR-targeting Inhibitors Block HIV-1 attachment and Infection in Raji-CD4-DCIR Cell Lines, Dendritic Cells and Apoptotic CD4TL

**Figure 5:**
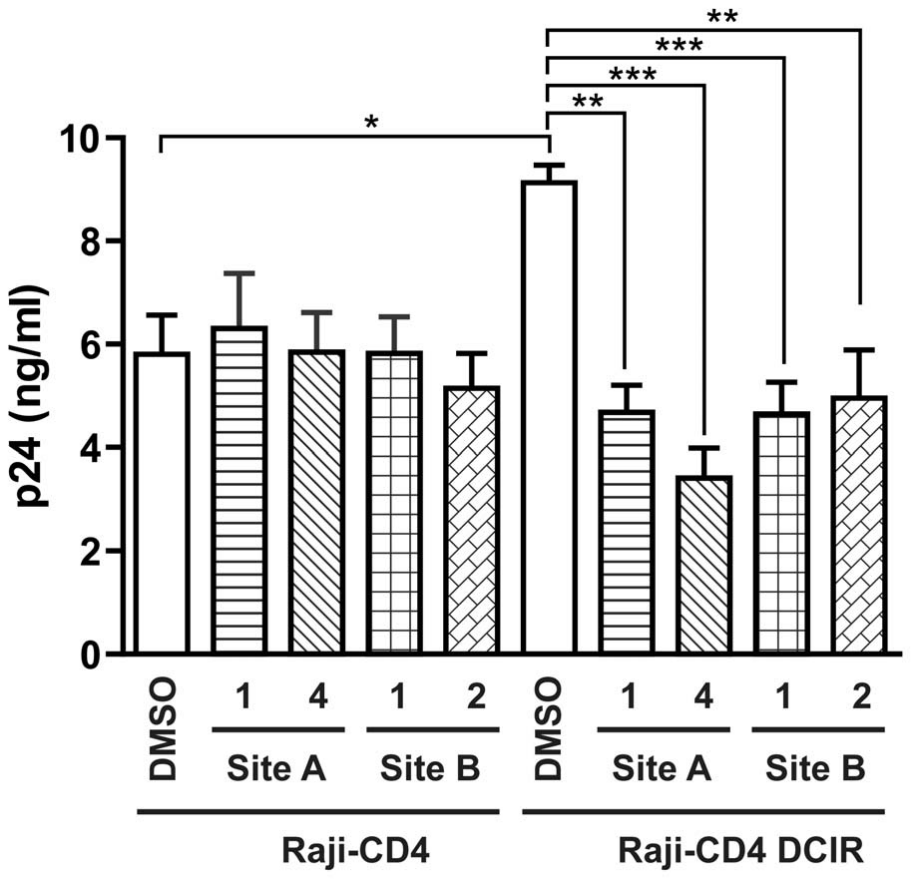
HIV-1 replication is diminished in DCIR-expressing cells by inhibitors specific for the site A and site B. Raji-CD4 and Raji-CD4-DCIR were treated with the inhibitors selected in figure 2, or DMSO. Cells were then exposed to NL4-3 for 2 h, rinsed and maintained in culture for 3d. Cell-free culture supernatants were collected and assayed for p24 content. Data shown correspond to the means ± SEM from 3 independent experiments performed with triplicate samples. Asterisks denote statistically significant values (*, *P*<0.05, ** P<0.01, *** P<0.001).

Since attachment to DCIR correlates with an increase in the infectivity of HIV-1, the impact of pre-treatment with the selected inhibitors on viral replication in Raji-CD4-DCIR cells was evaluated. Figure 5 shows that all four inhibitors decreased HIV-1 production in DCIR-expressing cells. None of the inhibitors had any effect on the replication of HIV-1 in Raji-CD4 cells, highlighting the specificity and the potency of the DCIR inhibitor. These data show that reduced HIV-1 attachment decreased viral infectivity measured after three days, following pre-incubation with the drug candidates. It should be noted that no significant inhibition was observed six or nine days post infection because the inhibitors were only added in a pre-treatment.

**Figure 6:**
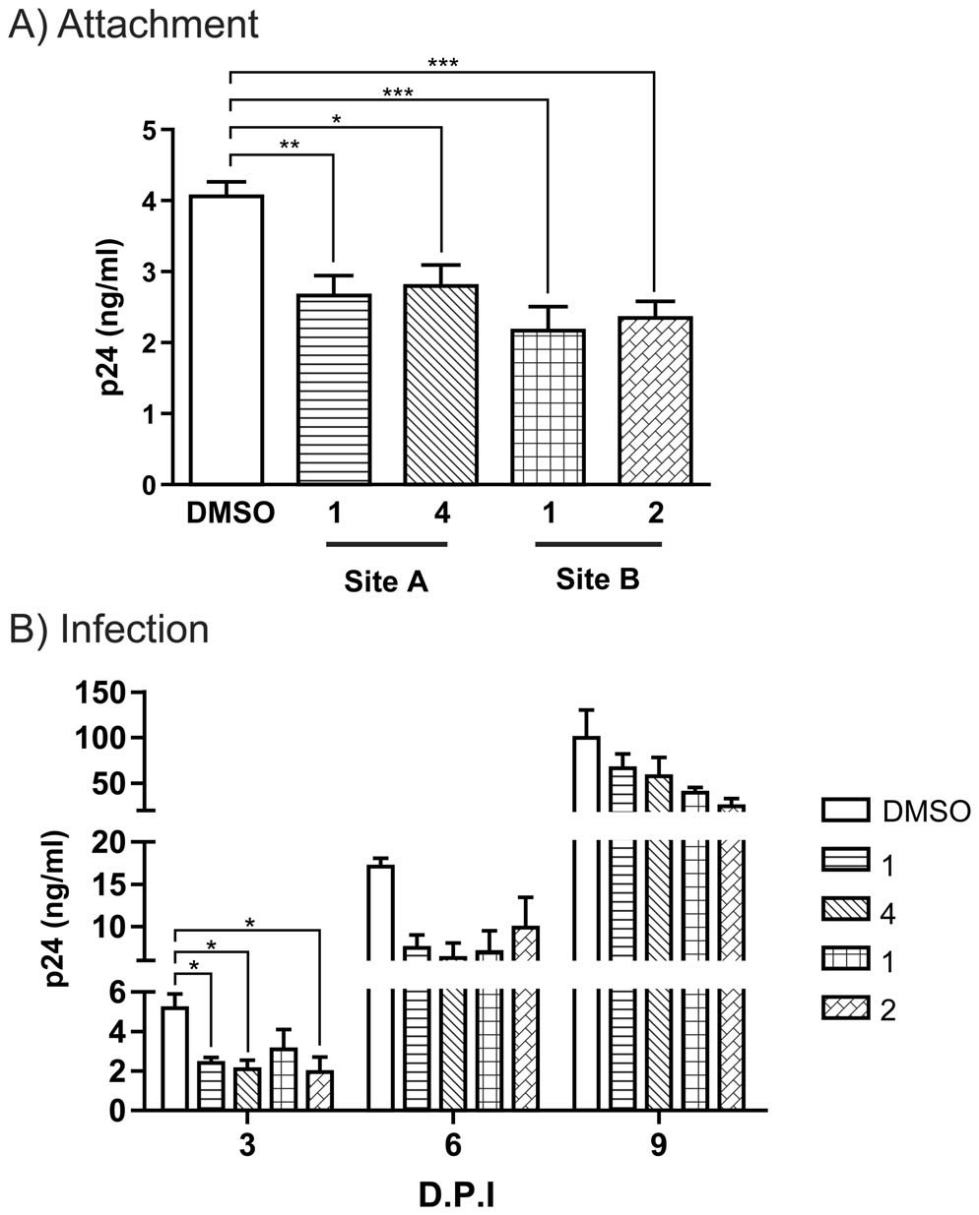
DCIR inhibitors reduce HIV-1 attachment and infection on primary dendritic cells. **A)** IM-MDDCs were treated with four chemical inhibitors or DMSO for 10 min at 37°C. Next, cells were pulsed with NL4-3bal*env* for 60 min at 37°C and rinsed thoroughly before measuring p24 content. **B)** In some experiments, similarly treated IM-MDDCs were pulsed with NL4-3bal*env* for 2 h at 37°C, rinsed thoroughly, and maintained in complete culture medium supplemented with GM-CSF and IL-4 for up to 9 days with medium replenishment every 3 days. Cell-free culture supernatants were quantified by measuring p24 content. Data shown correspond to the means ± SEM of 3 independent experiments performed in triplicate. Asterisks denote statistically significant values (*, *P*<0.05; **, *P*<0.01; ***, *P*<0.001).

After determining that the DCIR inhibitors blocked the interaction of HIV-1 with DCIR in cell lines expressing DCIR, we validated the activity of the selected inhibitors under more physiological conditions. The effects of the DCIR inhibitors on the attachment of HIV-1 and on cis-infection were tested using DCs. Figure 6A shows that HIV-1 binding to DCs pre-incubated with the four inhibitors was decreased significantly and that all four inhibitors also blocked productive infection at days 3 (figure 6B). As observed for Raji-CD4-DCIR (figure 5B), a new virions production at day 6 and 9 was not affected by pre-treatment with inhibitors.

**Figure 7:**
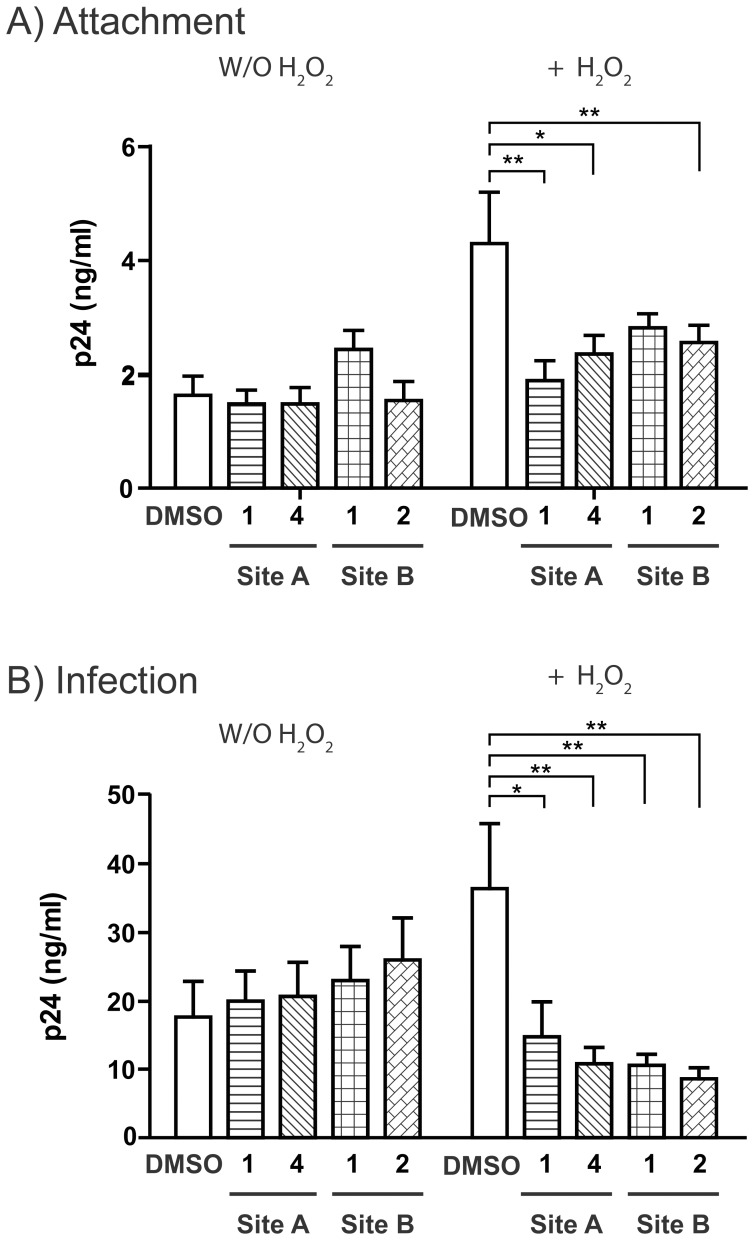
Impact of DCIR inhibitors on HIV-1 transmission by apoptotic CD4^+^ T cells. Target CD4^+^ T cells (1×10^6^) were treated for 16 h with H_2_O_2_ (30 µM) to induce apoptosis and the surface expression of DCIR. Cells (± H_2_O_2_ treatment) were incubated for 10 min with a site A inhibitor (1 and 4) or a site B inhibitor (1 and 2) or with 10 mM DMSO. A) Cells were next exposed to NL4-3 for 1 h at 37°C, washed thoroughly to remove un-adsorbed virions before assessing p24 content. B) Cells were first incubated with NL4-3 for 2 h at 37°C, washed thoroughly to remove un-adsorbed virions and cultured with autologous PHA-L/IL-2-activated CD4TL in complete RPMI-1640 supplemented with rhIL-2. Cell-free supernatants were collected on day 3 and assayed for p24 content. Data correspond to mean ± SEM of 4 independent experiments performed in triplicate for panel A and mean ± SEM of 4 independent experiments for panel B. Asterisks denote statistically significant values (*, *P*<0.05; **, *P*<0.01).

Pre-treatment of CD4TL with hydrogen peroxide (H_2_O_2_) mimics the apoptosis, a hallmark of evolution of AIDs. Expression of DCIR is also associated with this state and promote trans infection as we have shown previously [21]. We sought to confirm the role of DCIR in HIV-1 attachment (Figure 7A) and transmission (Figure 7B) via CD4TL by treating the cells with H_2_O_2_ before pre-incubation with the selected DCIR inhibitors (HIV-1 trans infection of PHA-L/IL2-activated CD4TL via apoptotic CD4TL). Figure 7A shows that A-1 decreased HIV-1 attachment to H_2_O_2_ treated cells by about 50%, while B-1 did so by about 35%. The inhibitors also lowered the propagation of HIV-1 by H_2_O_2_-treated CD4TL to PHAL/IL-2 activated CD4TL by 60–80% (Figure 7B). CD4TL proliferation is known to be important for HIV-1 replication and we have observed that the inhibitors did not affect cell proliferation (Figure 4). The levels of inhibition obtained with the compounds described in this study are comparable to those observed in our previous studies using antibody, siRNA, or intracellular inhibitors [17], [48]. DCIR is not the only surface molecule involved in HIV-1 attachment to and infection of DCs. We therefore do not expect to observe complete inhibition of DC infection.

### 3D Structure of DCIR and ADMET Potential for these Inhibitors

**Figure 8:**
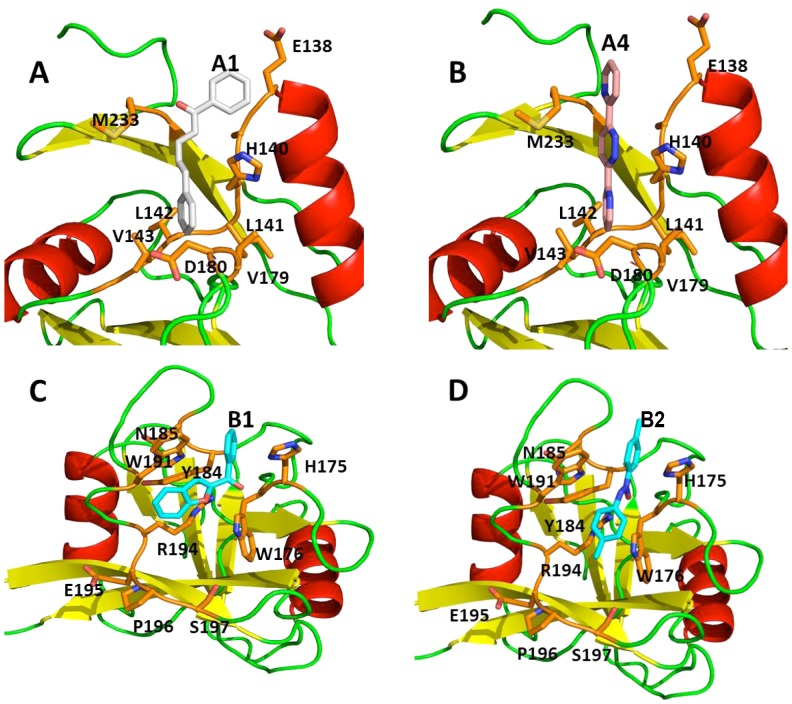
Three-dimensional schematic representation of the docking pose of selected active molecules. **Panel A**: Compound A1 (1,5-diphenyl-2,4-pentadien-1-one) in site A. **Panel B**: Compound A4∶3,6-di(2 pyridyl) pyridazine in site A. **Panel C**: Compound B1 (1-benzofuran-2-yl-phenylmethanone in site B. **Panel D**: Compound B2 (1-methyl-4-[(4-methylphenyl)-NNO-azoxy]benzene) in site B.

**Figure 9:**
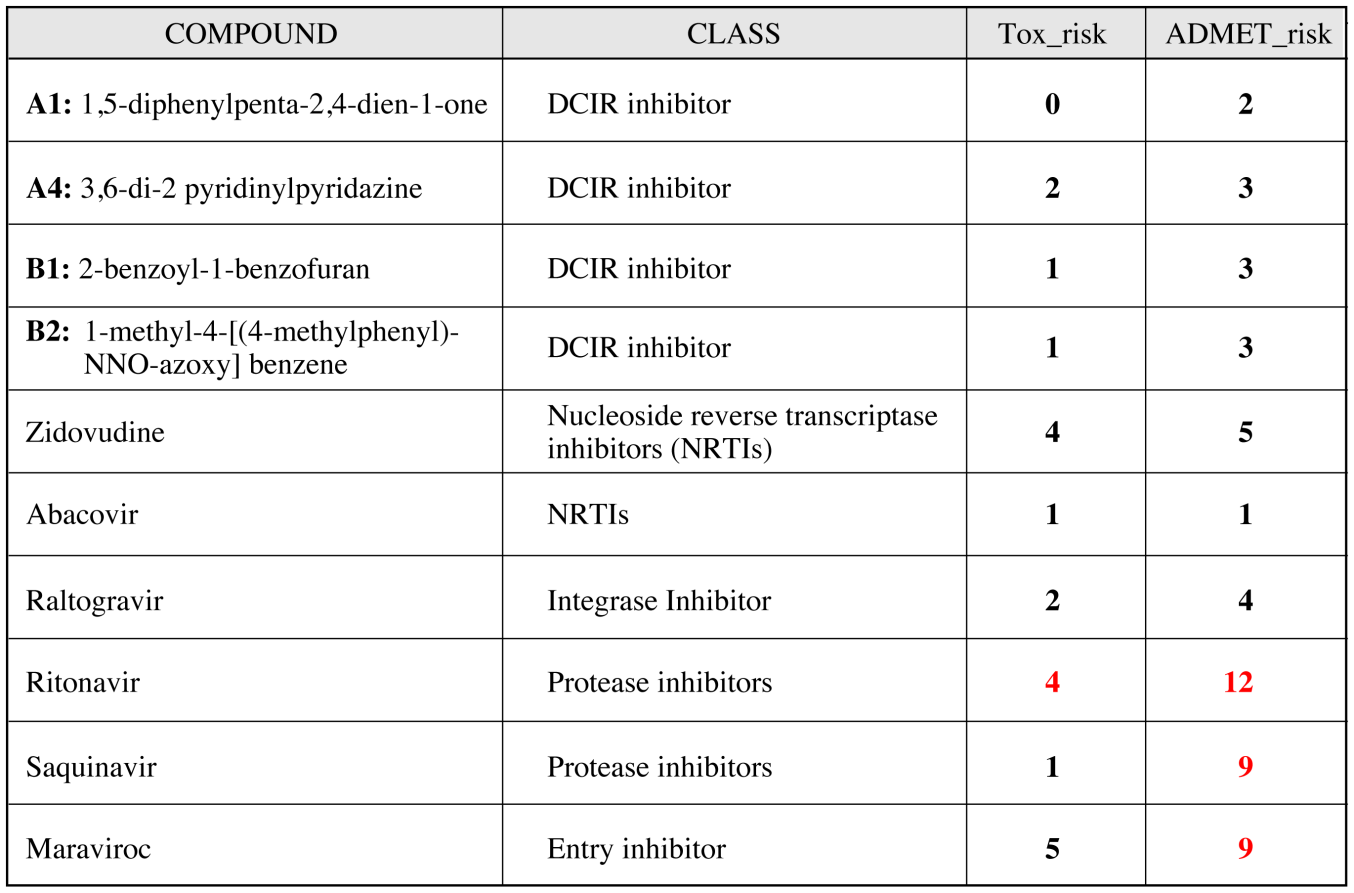
ADMET. Modelled ADMET (Absorption, Distribution, Metabolism, Elimination and Toxicity) properties of our first four lead compounds using ADMET predictor. These results shown that toxicity of our molecules is minimal compare to protease inhibitor or Maraviroc, both molecules currently used in clinic.

Figure 8 shows the probable orientation of compounds docking on the surface of the model of the DCIR molecule. Compound A1 and A4 dock in a pocket formed by Asn116, His140, Val143, Trp178, Asp180, Glu231 and Met233, while B1 and B2 dock in a pocket lined by residues His175, Trp176 Trp191, Arg194, Glu195, Pro196, Ser197, Tyr184 and Gln185. We have modelled the absorption, distribution, metabolism, elimination and toxicity (ADMET) properties of our four best compounds using ADMET predictor [53], [54], [55]. As illustrated in Figure 9, the toxicity and ADMET scores summarize the risks of low absorption from an oral dose, mutagenic activity, overall toxicity and metabolic liability. Low scores indicate low predicted toxicity or ADMET liability. Compared with the various classes of currently prescribed HIV-1 drugs, our compounds have lower scores in both risk assessments and are even better than most drugs in terms of ADMET properties. All these results are encouraging and justify future *in vivo* assay.

## Discussion

Despite great strides in our understanding of HIV-1 pathogenesis and immune protection, the pandemic keeps expanding while no effective cure appears likely to become available in the near future. Moreover, the anti-HIV-1 drugs developed so far promote the selection of resistant strains of the virus. The introduction of antiretroviral therapy in the mid 1990s had a strong impact on the course of HIV-1 infection throughout the world. Currently available treatments target different steps of the viral propagation cycle, such as entry into the host cell, reverse transcription, integration and protein maturation. These have led to significant reductions in HIV-related mortality [56]. Although combinations of antiretroviral drugs, such as HAART therapy, first met with resounding success, their limitations soon became obvious. Patient morbidity is enhanced and drug-resistant viruses have emerged, while no current antiretroviral therapy actually eradicates the virus from the body [57], [58].

Based on the major role played by DCIR in HIV-1 infection, we provide novel strategies to block HIV-1 transmission by DCs as well as by apoptotic or HIV-1-infected CD4TL. In this study, a detailed three-dimensional structure of DCIR has been proposed and four inhibitors directed against the CRD domain and EPS motif of DCIR blocking HIV-1 replication and propagation have been identified. These results are clinically relevant, since blocking HIV-1 attachment to DCIR may represent a novel strategy against HIV-1 pathogenesis. Indeed, preventing the virus from binding to DCIR could lead to a significant decrease of transmission during primary infection, a period during which the virus is disseminated by mucosal DCs expressing DCIR and ultimately transferred to CD4TL. In addition, DCIR inhibitors can reduce the production of HIV-1 by the CD4TL, therefore being useful in prophylaxis/primo infection and therapeutic stages of HIV-1 infection.

The discovery of new therapeutic targets and the development of new approaches to treatment are necessary in order to pursue the fight against HIV-1 [59], [60]. New classes of inhibitors targeting cellular partners of HIV virions are being developed [3], [61] including integrase inhibitor, antagonists of co-receptors CCR5 and CXCR4 (one is already commercially available), maturation process inhibitors, CDK inhibitors, anti-CD4 antibodies, and new attachment factor inhibitors such as anti-DC-SIGN antagonists [62], [63].

Molecules targeting DC-SIGN interfere with HIV-1 binding through interaction with viral gp120 [64], [65]. This mannose-binding lectin is expressed on cells in mucosal tissue and can thus facilitate HIV-1 transmission. Inhibitors of gp120/DC-SIGN interaction should therefore be useful primarily for preventing HIV-1 infection [66], since DC-SIGN is known to be involved only in trans-infection of DCs [18], [19]. The success of pre-exposure prophylaxis or PREP [67] validates the importance of acting early, as argued by Haase [7]. In spite of the intense competition for the HIV-1 drug market (Kalorama, 2008, *HIV/AIDS markets)*, the discovery of new targets and new molecules that do not select resistant forms of the virus and block X4 or R5 tropic virions continues to lag [68], [69]. Molecules targeting DCIR can be used to block the initial steps of infection and are effective against X4 or R5 as well. In addition, specific expression on infected CD4TL or apoptotic CD4TL makes DCIR an interesting potential target for treatment of infection.

In vitro tests of molecules targeting DCIR showed that four of five candidates selected by ChemScore ranking were active inhibitors of HIV-1 binding, indicating that our DCIR docking platform setting has interesting potential for improving the selection of inhibitors specific for the DCIR-HIV-1 interaction. Our virtual model and screening process closely matched the actual DCIR structure. We initially screened all inhibitors for their inhibitory activity against HIV-1 binding prior to performing in vitro experiments with Raji-CD4 transfected with either DCIR or vector only. Among the A series compounds (i.e. directed against the CRD domain), compounds A2 and A3 were only weakly inhibitory to HIV-1 binding, as was B3 (directed against the EPS motif), while A1, A4 and B1 and B2 were relatively strong inhibitors. Interestingly, the two active inhibitors differ in structure, are directed against different motifs and hence have specific sites of action, based on modeling. In addition, these inhibitors had no impact on expression of the HIV-1 co-receptors CXCR4 and CCR5 (data not shown). In vitro experiments with Raji-DC-SIGN, to evaluate the specificity of these inhibitors, confirmed that they do not block HIV-1 attachment to DC-SIGN. This latter C-type lectin is known to bind several types of viruses. Based on these results, we suggest that the inhibitors, thus selected, are very likely specific for the attachment of HIV-1 to DCIR. In addition, these selected compounds shows a predicted toxicity as measured by ADMET, better than the current prescribed drugs. These are confirmed at the cellular level by our toxicity test.

A physiologically relevant analysis of the activity of the inhibitors was carried-out using two major cell types involved in HIV-1 pathogenesis, DCs and CD4TL. The inhibitors displayed specificity for inhibiting HIV-1 attachment and subsequent infection in immature monocyte-derived dendritic cells (iMDDCs). We previously showed that some CD4TLs express DCIR in HIV-1 patients [21]. DCIR expression increases during the infectious process and promotes trans-infection and transmission of HIV-1 to bystander cells. DCIR appears specifically linked to the infectious process and to cell apoptosis. This is important, since previous studies suggest a direct correlation between the degree of apoptosis among circulating CD4TLs and pathogenesis [70], [71].

The results of the present study demonstrate that the selected DCIR-targeting inhibitors are effective in this model resembling the chronic phase of HIV-1 infection. Based on our preliminary observations, we can predict that inhibitors designed to bind specifically to the CRD domain and EPS motif of DCIR can decrease HIV-1 attachment to DCs in primary infection and result in reduced DC infection.

In developing novel therapeutic drugs against HIV-1 infection, one must consider the mechanism of viral replication not only inside CD4TLs, but also inside DCs. We agree with Haase (3) and other that it is crucial to find appropriate ways and means to alleviate viral load and allow the proper mounting of specific immune responses, both in the crucial early phases to avoid irreversible damage to the immune system and during the chronic phase to allow the immune system to mount effective defences. Future design of new drugs against HIV-1 infection should focus on preventing irreversible impairment of the immune system. Indeed, currently used anti-HIV-1 drugs block only the late stages of the viral life cycle and provide no protection against the earlier damage and resulting immunodeficiency that it causes. By targeting the C-type DCIR lectin found in DCs as well as in HIV-1-infected CD4LTs, the approach described in this report provides a potential avenue for effective interference with the initial propagation of HIV-1 at an early stage of the viral cycle and limit proliferation of the virus in the later stages. Extensive in vivo validation nevertheless remains to be performed in order to validate the targeting of DCIR as a therapeutic approach, as well as the safety of the drugs used for this purpose.
